# Behavior in the elevated plus maze is differentially affected by testing conditions in rats under and over three weeks of age

**DOI:** 10.3389/fnbeh.2015.00031

**Published:** 2015-02-17

**Authors:** Sarah H. Albani, Marina M. Andrawis, Rio Jeane H. Abella, John T. Fulghum, Naghmeh Vafamand, Theodore C. Dumas

**Affiliations:** Department of Molecular Neuroscience, Krasnow Institute for Advanced Study, George Mason UniversityFairfax, VA, USA

**Keywords:** elevated plus-maze, anxiety, postnatal development, environment, corticosterone, *Arc*, immediate early-gene, juvenile

## Abstract

The late postnatal period in rats is marked by numerous changes in perceptual and cognitive abilities. As such, age-related variation in cognitive test performance might result in part from disparate sensitivities to environmental factors. To better understand how testing conditions might interact with age, we assessed anxiety behavior on an elevated plus maze (EPM) in juvenile rats around 3 weeks of age under diverse testing conditions. Plasma corticosterone and neuronal activation patterns in the forebrain were examined after maze exposure. We found that anxiety was differentially expressed during different stages of late postnatal development. Bright illumination and morning testing encouraged greatest open arm exploration on the EPM in younger animals, while older rats explored open areas more under dim illumination in the morning compared to bright illumination in the afternoon/evening. Older rats exhibited higher plasma corticosterone levels at baseline compared to younger rats; however, this trend was reversed for post-testing corticosterone. Additionally, post-testing corticosterone levels were inversely related to time of testing. Compared to testing in the morning, EPM exposure in the afternoon/evening elicited greater neuronal *Arc* expression in the amygdala. *Arc* expression in the amygdala after morning testing was greater at P22–24 than P17–19. In layer 2/3 of primary visual cortex, *Arc* expression was elevated in younger animals and age interacted with time of testing to produce opposing effects at P17–19 and P22–24. These data suggest that age-related differences in anxiety-associated behavior during the late postnatal period are due in part to changes in light sensitivity and emergence of a circadian cycle for corticosterone. The findings illustrate that late postnatal behavioral development in rodents is a complex orchestration of changes in neural systems involved in perception, cognition, affect and homeostatic regulation.

## Introduction

Perceptual and cognitive abilities in rats undergo substantial postnatal modification between the second and fourth weeks of life. While being tended for in the nest, the rat pup hormonal stress response to most environmental stimuli is blunted (Vázquez, 1998), focusing physiological responses to interactions between offspring and dam. At around 2 weeks of age, rat pups exit this stress hyporesponsive period and are more sensitive to environmental stimuli (Sapolsky and Meaney, 1986; Widmaier, 1990) as they begin to explore outside of the nest. Also during this time, more distal sensory capacities such as vision and audition start to mature and reach adult status in the fourth postnatal week (Crowley and Hepp-Reymond, 1966; Hyson and Rudy, 1984; Fagiolini et al., 1994; Morishita and Hensch, 2008). In parallel, activity-rest cycles and the circadian rhythm for the major stress hormone, corticosterone (CORT), begin to shift in response to light during the third postnatal week and are not different from adult cycles by the fourth postnatal week (Reppert et al., 1984; Honma and Honma, 1986; Rosenfeld et al., 1992). The comprehensive alterations in physiological and behavioral processes that occur during the late postnatal period represent a qualitative change in how the organism interacts with its environment and suggest that performance differences in tasks designed to investigate cognitive maturation near 3 weeks of age may be influenced by developmental changes in basic perceptual skills, homeostatic regulation, or affective responses to environmental conditions. Therefore, it is important to better understand how testing conditions impact innate anxiety and risk assessment in subjects under and over three postnatal weeks of age.

Prominently, excitatory synaptic transmission in the forebrain continues to mature throughout the first month after birth (Dumas, 2005; Stoneham et al., 2010; Cooper and Bear, 2012). The major glutamatergic ionotropic receptors, α-amino-3-hydroxy-5-methyl-4-isoxazolepropionic acid receptors (AMPARs), undergo structural and functional modification during late postnatal development (Durand and Zukin, 1993; Standley et al., 1995; Martin et al., 1998; Dingledine et al., 1999 review) along with changes in exploratory behavior (Douglas et al., 1973; Dumas, 2004). Direct implication of alterations in AMPAR function in the developmental emergence of spatial cognition was recently shown via acute delivery of the positive allosteric modulator of AMPARs, CX614 (Arai and Kessler, 2007), during the same developmental stage examined in the present study (Blair et al., 2013). Enhanced exploration produced by pharmacological modulation of AMPARs could be the result of drug effects on developing sensory systems, affective circuits, or spatial navigation networks. Specific perceptual, affective, and cognitive affects of drug delivery in juvenile rats may be resolved by testing animals in different mazes under varied testing conditions.

The elevated plus maze (EPM) is used to assess innate anxiety in rodents (Handley and Mithani, 1984; Ohl et al., 2001; Walf and Frye, 2007). While extensively validated (Pellow et al., 1985; Walf and Frye, 2007), EPM studies often produce inconsistent findings, which may be attributed in part to methodological differences, including age, strain, or sex of the subjects and housing or testing conditions (Nagy and Glaser, 1970; Rodgers and Dalvi, 1997; File, 2001; Andrade et al., 2003; Walf and Frye, 2007). To some extent, subject age has been investigated (Imhof et al., 1993; Andrade et al., 2003; Doremus et al., 2006; Lynn and Brown, 2010), but only one study included a time point under 1 month of age and this group was compared to adults at 2 months (Smith and Morrell, 2007), limiting the resolution of the developmental time course for innate anxiety. Similarly, lighting conditions have been examined in adult animals, but most studies compare between light and dark cycles, such that lighting condition is confounded by circadian period (Andrade et al., 2003), and to our knowledge, only one study has looked at circadian influences strictly within either the light or dark period (Griebel et al., 1993). Without proper parametric analysis of developmental changes in EPM performance, it is not possible to accurately assess age-related alterations in innate anxiety or risk assessment.

To better characterize impacts of biological and environmental variables on EPM performance, we systematically varied age of testing, time of testing, and lighting level during testing within the circadian light cycle. We also included pharmacology experiments using CX614 to tease out synaptic factors that might underlie any behavioral alterations. Rats were exposed to the EPM in the second or third postnatal week of age, under bright or dim illumination, in the morning (AM) or afternoon/evening (PM) and after delivery of CX614 or vehicle. Plasma CORT levels were measured to test for age-related differences in stress responsiveness. Expression levels of the activity-dependent immediate early gene,* Arc*, were quantified in the amygdala, primary visual cortex (V1), and hippocampus to gain some insight into neural representations of fear, sensitivity to lighting, and spatial encoding, respectively. The results indicated no effect of AMPAR modulation on EPM behavior, plasma CORT level, or neural activation in select brain regions at either testing age. However, environmental variables differentially influenced behavioral performance, plasma CORT responses, and neuronal activation according to age. Overall, this study reveals endocrine and neural regulators of anxiety-like behavior during an important period of postnatal forebrain and cognitive maturation in rats.

## Methods

### Subjects

Long-Evans rats were bred in the Krasnow Institute Animal Facility and housed in individually ventilated cages in a temperature- and humidity-controlled vivarium under a 12:12 h light/dark cycle (lights on at 0700 h). Female breeders were drawn from the colony while male breeders were imported (Charles River Labs, Frederick, MD). Food and water were available *ad libitum*. Pups were not weaned until completion of behavior testing. Rats were maintained and handled in accordance with the regulations stated in the *Guide for Care and Use of Laboratory Animals* by the National Research Council. The George Mason University Institutional Animal Care and Use Committee (IACUC) approved all procedures employed.

Rats were pseudorandomly assigned to experimental groups, with each group reflecting an age, time of day (TOD) for behavior testing, lighting during behavior testing, and CX614 dosage assignment. Litters were evenly divided such that half of the pups were tested at postnatal day (P) 17–19, while the remaining half were tested at P22–24. Time of day (TOD, AM or PM) and lighting conditions (dim or bright) were varied across litters, but remained consistent within litters.

### Behavioral testing

#### Apparatus and setup

The EPM was constructed from pine boards, painted black and sealed with polyurethane. It consisted of two opposing open arms (length: 40 cm, width: 9 cm) and two opposing wall-enclosed arms (wall height: 15 cm) extending off of a center square (9 cm per side) to form a plus shape. The maze was placed on a pedestal (70 cm high) in the center of a rectangular room (3 × 3.7 meters) with white walls containing large black painted shapes. A video camera was hung from the ceiling (2.7 meters high), directly above the center of the maze.

The dim illumination condition was produced by two tall floor lamps with translucent shades placed at opposite corners of the maze. The total luminosity, as measured at the end of each open arm, was 400 lux. The bright illumination condition included additional florescent ceiling lights, creating a luminosity of 1200 lux on the open arms. AM testing was performed between 0700 h and 1200 h, while PM testing was performed from 1300 h to 1900 h. Assignment to lighting and TOD testing conditions was counterbalanced as much as possible for each testing age range, within each litter. We confirmed that the TODs for the AM and PM groups were statistically different overall (independent samples *t*-tests: *t*_(181.98)_ = 22.19, *p* < 0.001), and for each age group individually (P17–19: *t*_(103.87)_ = 16.99, *p* < 0.001; P22–24: *t*_(73.61)_ = 14.47, *p* < 0.001).

Cyclodextrin (1 g in 1 mL sterile saline plus 1 mL sterile water) was used as a vehicle for delivery of the AMPAR allosteric modulator, CX614 (Cortex Pharmaceuticals, Irvine, CA). CX614 was freshly dissolved in cyclodextrin (Sigma Aldrich) at the start of each behavior testing session at a 4X dilution to deliver final dosages of 2.5 (*n* = 88) or 4.0 (*n* = 78) mg/kg injected intraperitoneally. Control rats were administered the vehicle alone (*n* = 109).

#### Testing procedure

Male and female rats were tested at P17–19 or P22–24. The home cage with all littermates and dam was transported from the housing room to the testing room 15 min prior to the start of the testing session. After acclimating for 10 min, the first animal was marked at the base of the tail with indelible ink, weighed, and administered either vehicle or CX614 inside of the testing room and returned to the home cage, which remained in the testing room. Testing order was counterbalanced across doses. EPM exposure began at 30 min post-injection and lasted for 8 min. Each rat was placed in the center of the EPM facing an open arm and then the experimenter exited the room. The maze was cleaned with 70% ethanol between runs to minimize scent trails. Each animal underwent only one maze exposure and was then euthanized to maintain TOD and lighting conditions across behavioral and biological assays.

### Corticosterone (CORT) assay

Five minutes after completion of the maze test, animals were anesthetized with Isoflurane vapor (>5%). After severing the renal artery, volumes of 0.5 mL to 1.5 mL of blood released from rat kidney were collected in 2 mL centrifuge tubes containing heparin (10 uL, 2 mg/mL in sterile water). Heparinized blood samples were centrifuged (10 min, 14,000 rpm, 4°C) within 15 min after collection and plasma was isolated and stored at −20°C until assayed. Although blood was collected from the majority of animals tested, samples were selected for assay on the basis of adequate volume, clear plasma color, and representation among each TOD and Age group combination. Samples were diluted 1:10 in Tris buffered saline and CORT levels were assayed by enzyme-linked immunosorbent assay (ELISA) according to the manufacturer’s protocol (Corticosterone EIA Kit, Enzo Life Sciences, USA). Optical density readings were taken at 405, 570, and 590 nm for each plasma sample using Synergy 4 Microplate reader (BioTek, USA) and standardized to the average value of the blank wells within each plate. Standardized CORT concentration for each animal was normalized by dividing by the mean CORT level calculated for EPM naïve control animals matched for age and TOD (collections were taken at 0800 h for AM controls and 2000 h for PM controls). Control animals did not experience the EPM but were matched to experimental animals for TOD and age.

### *In situ* hybridization

Following renal blood collections, pericardial perfusion was performed with 4% paraformaldehyde (PFA) in phosphate buffered saline (1X PBS). After full perfusion, brains were extracted, post-fixed overnight in 4% PFA and then stored in 30% sucrose at 4°C prior to sectioning. Brains were sectioned in the sagittal plane with a cryostat (30 μm), adhered to glass slides (SuperFrost Plus, Fisher), and stored at −80°C for subsequent *in situ* hybridization (ISH).

Colorimetric whole mount ISH was performed on a sample of brain tissue sections representative of varying ages and environmental testing conditions. Best quality brain sections were selected from each EPM testing group prior to reaction (unbiased with respect to *Arc* expression). Each individual reaction contained equal representation tissue sections across variables (Age, TOD, and Illumination). An effort was made to select tissue sections from animals whose blood was also selected for CORT analysis. Antisense riboprobe (single strand, ~3000 bp) incorporating digoxygenin-labeled uracil nucleotides was transcribed from a linearized DNA template containing rat *Arc* cDNA (generous gift from Dr. Paul Worley) and purified by spin column (E.Z.N.A, USA). Riboprobe was hybridized to the tissue sections at 62°C overnight. Sections were subsequently washed in 50% Formamide/1X Saline Sodium Citrate, maleic acid buffer with 0.1% Tween 20 (MABT) to remove unbound probe and prepare the tissue for antibody labeling. Sections were incubated overnight at room temperature in an alkaline-phosphatase (AP) conjugated anti-digoxigenin primary antibody (Roche Applied Science, USA). Following washes in MABT and AP Staining Buffer, slides were developed in NBT/BCIP for about 4 h, or until purple staining was apparent. Slides were then dehydrated through sequential washes in increasing ethanol concentration, defatted with xylenes, and coverslipped. Brightfield images were collected at 2X magnification. The number of *Arc*-positive neurons in layers 2/3 and 4 of the primary visual cortex (V1), area CA1 of the dorsal hippocampus, and the basal, lateral, medial and central nuclei of the amygdala were counted by investigators blind to experimental condition using the particle analysis feature of ImageJ software (Rasband, W.S., NIH), along with the aid of a rat brain atlas (Paxinos and Watson, 2007) as a guide for anatomical specificity. First, a background level was established by selecting an area of tissue void of labeling, in close proximity to the region of interest. The auto-threshold option was then used to pull out features (i.e., cells) against this background setting. A unique background level was set for each anatomical area and, in some cases, the threshold level was further manually adjusted to ensure that the area where background was established did not yield any active features. Afterward, a polygon was drawn conforming to the boundaries of the region of interest and the particle analysis option was used to quantify the number of active particles within that area. Analysis areas were region-specific and were the same size for each anatomical structure across experimental groups. When calculating positive cell density, depth of sampling was assumed to be the sectioning thickness. Cell counts are presented as number of cells per given volume.

### Statistical analyses

Animal movements and regional dwell times were extracted electronically from each behavior video (TopScan software, CleverSys, Reston, VA). Time spent on each arm of the maze and in the center square was measured and arm entries were counted. Analysis of time spent in the open arms alone yielded low numbers and included many zero values, impeding data analysis. Therefore, we present open arm time as the sum of time spent on the open arms and time spent in the center square.

ANOVAs were performed for each of the behavioral, endocrine and neural indices. Four-way univariate ANOVAs (Age × Drug Dose × TOD × Lighting) were first conducted, then subsequent analyses were run to examine significant interaction effects. Bonferroni and Tukey HSD *post hoc* correction was applied to all applicable statistical tests. *T*-tests corrected for multiple comparisons were also used to compare single behavioral parameters of interest after higher-level analyses. Simple linear regression analyses were performed to analyze developmental influences on the relationships of testing time and CORT concentration as well as *Arc* expression and CORT level. Lastly, modified ANOVA interaction analyses were performed to further compare regression lines across ages. Outlying data points were excluded from analysis based on a calculation of three-standard deviations from the mean. Significance was determined at *p* < 0.05 for all tests. Statistical tests were performed using IBM SPSS Statistics software (IBM Corp., USA) and Microsoft Excel. All figures show group means. Error bars reflect one standard error of the mean.

## Results

### Elevated plus maze performance is impacted by developmental stage and testing conditions

**Figure 1:**
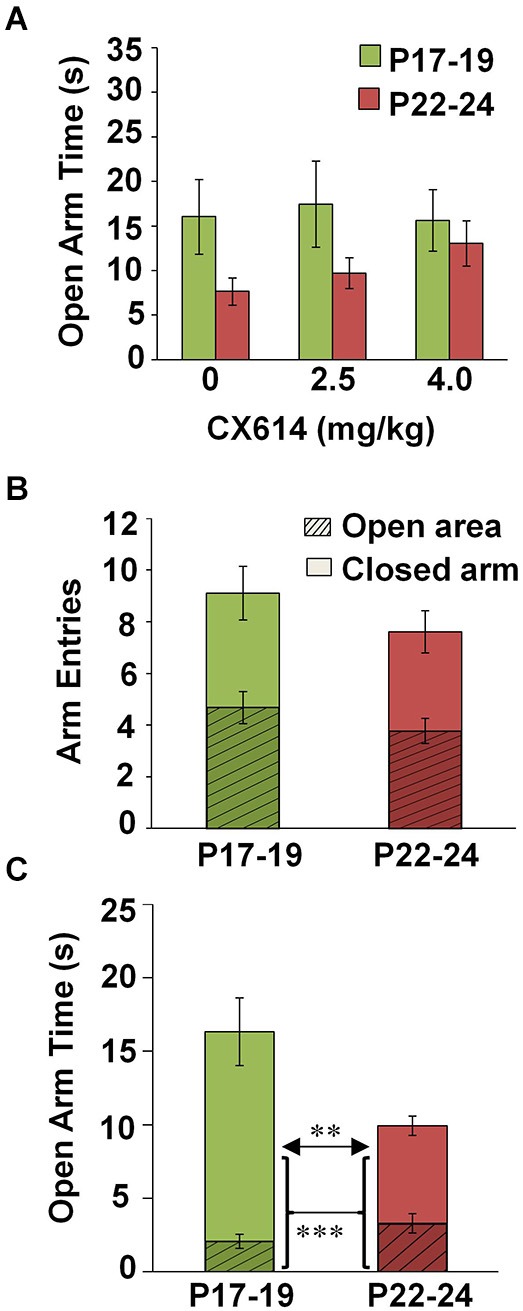
**Effects of CX614 and developmental stage on behavioral parameters of the EPM. (A)** Impact of CX614 on time spent in an open area. **(B)** Entries into enclosed and open areas at P17–19 and P22–24. Hatched-line regions represent the proportion of total arm entries that were made into an open area, while solid bars indicate the proportion of total entries into closed arms. **(C)** Dwell time in open arms separated out by open arm and center zone. Hatched-line bars represent the proportion of open arm time spent navigating open arms, while the solid portion of the bar indicates the proportion of time made up by center zone exploration. (P17–19: *n* = 112; P22–24: *n* = 145). ***p < 0.001, **p < 0.01.

To guide analysis of this multifactorial project, we first performed a four-way ANOVA comparing all variables (Age × TOD × lighting × Drug Dosage) with respect to open arm dwell time. This test revealed main effects of Age (*F*_(1,233)_ = 11.723, *p* = 0.001) and TOD (*F*_(1,233)_ = 9.549, *p* = 0.002). Additionally, interactions were found between Age × Lighting (*F*_(1,233)_ = 16.141, *p* < 0.001), Lighting × TOD (*F*_(1,233)_ = 7.516, *p* = 0.007), and Age × Lighting × TOD (*F*_(1,233)_ = 10.075, *p* = 0.002). There were no main effects of Drug on open arm time, or any interactions with other variables (Age × Drug, TOD × Drug, Lighting × Drug), (Figure 1A). As such, data in subsequent analyses were collapsed across drug conditions. Table 1 indicates the number of subjects per individual condition.

**Table 1:** **Number of subjects in each testing condition**.

	P17–19			P22–24		
	Behavior					
	Lighting			Lighting	
TOD	Dim	Bright	Totals	Dim	Bright	Totals
**AM**	26	18	44	56	34	90
**PM**	56	22	78	40	23	63
**Totals**	82	40	122	96	57	153
	**CORT**			***Arc***		
	**P17–19**	**P22–24**	**Totals**	**P17–19**	**P22–24**	**Totals**
**AM**	9	11	20	7	6	13
**PM**	5	8	13	8	11	19
**Totals**	14	19	33	15	17	32

*Number of animals per assay broken down by age group and further organized by TOD and lighting conditions*.

#### Open arm exploration on the EPM is increased at P17–19 compared to P22–24

We found no difference in the number of open arm entries nor total arm entries between animals tested at P17–19 (*n* = 122) and P22–24 (*n* = 153) (Figure 1B). However, a significant main effect of Age on open arm time was observed (four-way ANOVA: *F*_(1,233)_ = 11.723, *p* = 0.001), reflecting more time exploring open arms at P17–19 compared to P22–24. This effect was mainly due to greater time spent exploring the center square (independent samples *t*-test, *t*_(255)_, *p* = 0.002) as open arm time alone (minus the center square) was not different between groups (Figure 1C). These data suggest increased expression of innate anxiety with increasing age at the end of the third postnatal week.

#### Bright illumination reduces anxiety in the AM at P17–19 but increases anxiety behavior in the PM at P22–24

**Figure 2:**
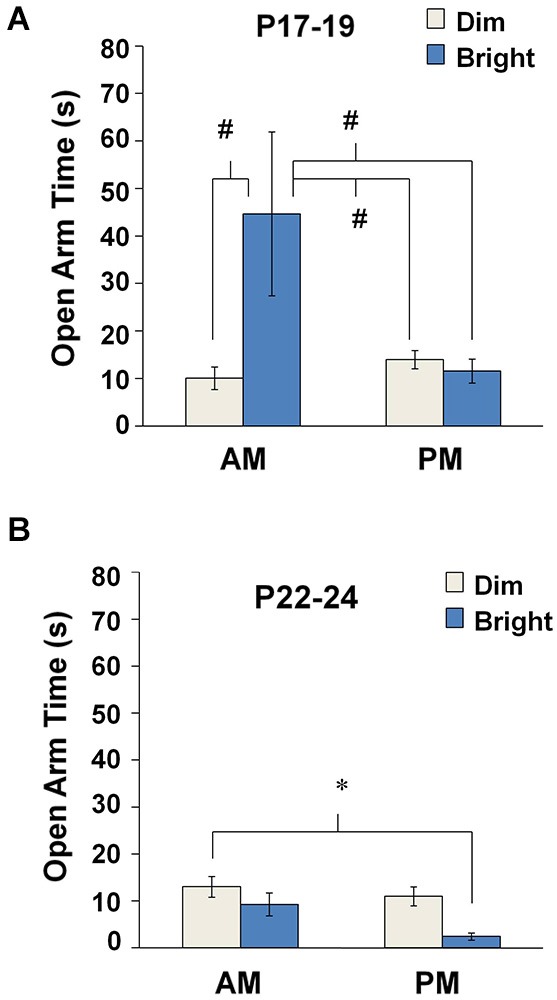
**Anxiety-like behavior in the EPM under different environmental parameters**. Mean open arm time for each testing condition at **(A)** P17–19 and **(B)** P22–24. #*p* < 0.001 compared to the bright-AM group, *p < 0.05.

Effects of testing conditions on open arm dwell time were analyzed separately at P17–19 and P22–24. At P17–19, we observed main effects of TOD (two-way ANOVA: *F*_(1,108)_ = 7.715, *p* = 0.006), lighting (*F*_(1,108)_ = 9.422, *p* = 0.003), as well as a TOD × lighting interaction (*F*_(1,108)_ = 12.758, *p* = 0.001). *Post hoc* analyses indicated that, at P17–19, open arm time was increased during bright illumination in the AM compared to all other groups (Bonferroni: dim-AM, *p* < 0.0001; dim-PM,* p* < 0.0001; bright-PM, *p* = 0.001) (Figure 2A). At P22–24, we observed a significant main effect of Illumination (*F*_(1,145)_ = 6.750, *p* = 0.01), but no significant main effect of TOD or a TOD × Illumination interaction. At P22–24, open arm time during bright illumination in the PM was significantly reduced compared to dim illumination in the AM (Bonferroni: *p* = 0.017) (Figure 2B). Combined, these findings support an ability of bright illumination to heighten innate anxiety in animals over 3 weeks of age, especially in the AM.

### Developmental stage and testing conditions influence stress reactivity to EPM exposure

#### Baseline plasma CORT increases from P17–19 to P22–24

**Figure 3:**
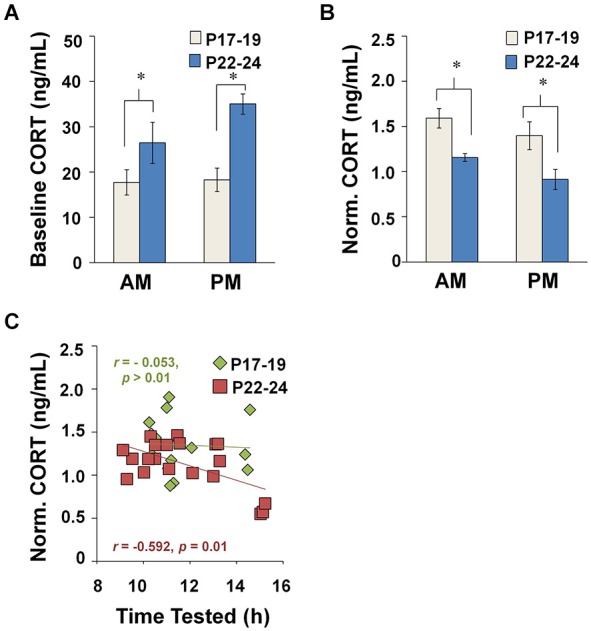
**Plasma CORT concentrations measured at baseline and after EPM exposure. (A)** Baseline CORT levels during diurnal AM and PM. 4–7 animals were represented in each TOD category per age group. **(B)** Impact of TOD and age on CORT levels in maze-exposed rats (P17–19: AM, *n* = 9; PM, *n* = 6; P22–24: AM, *n* = 13 and PM, *n* = 15). CORT concentrations were higher after testing at P17–19 compared to P22–24, regardless of whether tested during the AM (Tukey HSD: *p* = 0.012) or PM (Tukey HSD: *p* < 0.001). **(C)** CORT in maze-exposed rats plotted against time of testing. *p < 0.05.

Immunoassays for plasma CORT were performed on blood plasma samples collected from behaviorally naïve controls in the AM or PM at P17–19 (*n* = 10) or P22–24 (*n* = 13) (Figure 3A). A two-way ANOVA showed that baseline CORT levels were elevated at P22–24 compared to P17–19 (*F*_(1,19)_ = 8.861, *p* = 0.008). There was no effect of TOD and no Age × TOD interaction. These results point to a developmental increase in baseline CORT at the end of the third postnatal week.

#### Stress reactivity to EPM exposure is influenced by TOD at P22–24, but not P17–19

Data from EPM-tested animals (P17–19: *n* = 25, P22–24: *n* = 32) were normalized to the mean baseline values from the appropriate age and TOD-matched control animals. Like the experimental animals, these controls underwent transport, handling, and exposure to a novel room, but were not exposed to the EPM. We performed a three-way ANOVA on normalized CORT level to detect any effects of Age, TOD and Drug Dose (collapsed across illumination conditions due to low *n* values). We found a main effect of Age (*F*_(1,22)_ = 11.364, *p* = 0.003), a near significant effect of TOD (*F*_(1,22)_ = 3.434, *p* = 0.077), and no significant interaction effects. No effect of CX614 treatment was found; therefore, data in subsequent CORT analyses were collapsed across drug conditions.

A two-way ANOVA revealed a main effect of Age (*F*_(1,30)_ = 17.655, *p* < 0.001) and a near significant effect of TOD (*F*_(1,30)_ = 3.654, *p* = 0.066), with no interaction effects between these variables. CORT levels were elevated after maze testing more at P17–19 than at P22–24, regardless of whether testing was in the AM (Tukey HSD: *p* = 0.012) or PM (Tukey HSD: *p* < 0.001) (Figure 3B). These data suggest that the CORT response to the EPM becomes less robust across the developmental period investigated.

Linear regression analyses revealed an inverse relationship between CORT level and TOD at P22–24 (Pearson correlation: *r* = −0.585, *n* = 18, *p* = 0.010), but not P17–19. Additionally, the regression lines were statistically different across age groups (two-way ANOVA interaction analysis: *t*_(36)_ = 3.366, *p* < 0.0025) (Figure 3C). These data suggest that age differences in the CORT response to EPM exposure arise, in part, due a developmental emergence of a diurnal rhythm for CORT after 3 weeks of age.

### Neuronal activation levels produced by EPM exposure differ across developmental stages and environmental conditions in amygdala and visual cortex, but not the hippocampus

#### Baseline Arc expression differs according to age in the hippocampus

**Figure 4:**
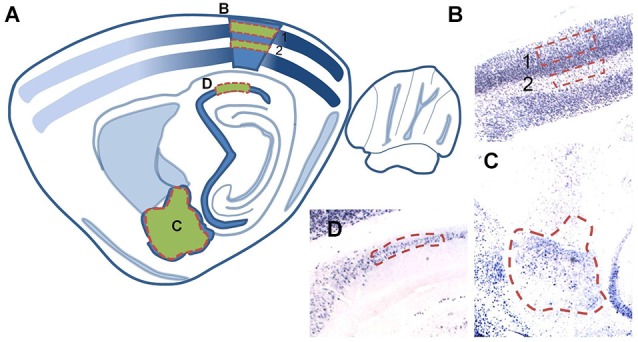
**(A)** Schematic illustration of a sagittal brain section (adapted from Paxinos and Watson, 2007) denoting regions in which *Arc* expression was quantified. Regions used for cell counting analyses are shaded green with red dashed outlines. **(B,C)** Corresponding brightfield images of tissue sections (2X magnification) of individual analysis regions: **(B)** layer 2/3 (1) and layer 4 (2) of area V1, **(C)** the basal, lateral, medial, and central nuclei of the amygdala and **(D)** area CA1 of the hippocampus.

**Figure 5:**
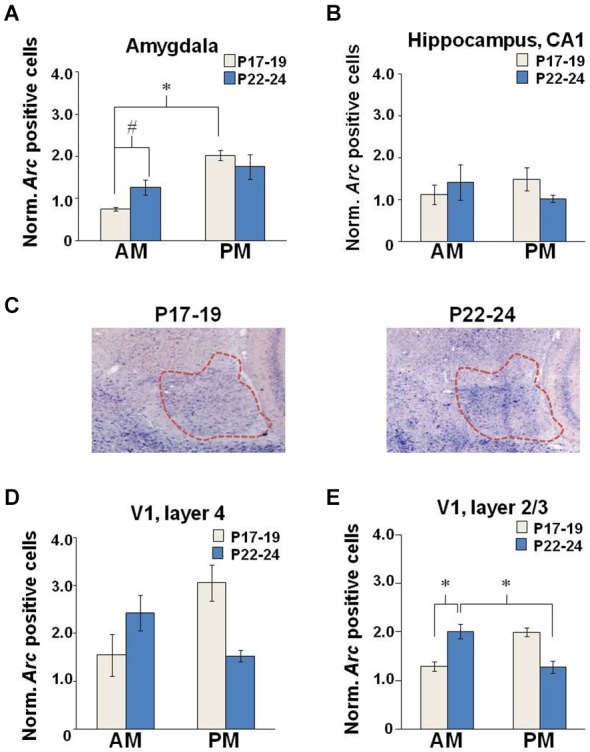
**Neuronal** ***Arc* expression after EPM exposure**. Quantification of cell counts normalized to baseline levels in the **(A)** amygdala, **(B)** hippocampus, and V1 **(D)** layer 4 and **(E)** layer 2/3. **(C)** Brightfield images (2X magnification) illustrating *Arc* intensity in the amygdala (outlined in red) in young and older developing rats tested in the PM. (P17–19: AM, *n* = 9; PM, *n* = 6; P22–24: AM, *n* = 13 and PM, *n* = 15), *p < 0.05, #*p* = 0.053.

Expression levels for the activity-dependent immediate early gene, *Arc*, (Guzowski et al., 1999; Ramírez-Amaya et al., 2005) were measured in brain regions associated with anxiety-like behavior (the amygdala, Gray and McNaughton, 2000; LeDoux, 2000), visual perception (cortical area V1, McCurry et al., 2010), and spatial navigation (hippocampus, Guzowski et al., 2001). First, ISH was performed on brain sections collected from behaviorally naïve control animals (P17–19: *n* = 7; P22–24: *n* = 9). A two-way ANOVA (Age × TOD) revealed a main effect of Age on the number *Arc* positive cells in area CA1 of the hippocampus (*F*_(1,15)_ = 6.026, *p* = 0.026), in addition to an Age × TOD interaction (*F*_(1,15)_ = 12.936, *p* = 0.003). At P17–19, *Arc* expression was greater in the AM than PM (Tukey HSD: *p* = 0.035). Likewise, *Arc* expression at P17–19 in the AM was greater than measured at P22–24 in the AM (Tukey HSD: *p* = 0.008). Also, there were Age × TOD interaction effects for layer 2/3 (*F*_(1,14)_ = 7.589, *p* = 0.015) and layer 4 (*F*_(1,14)_ = 7.524, *p* = 0.016) of V1. Due to difficulty in establishing subregional borders within the amygdala of younger animals, quantification of *Arc* in the amygdala encompassed the lateral, basolateral, medial and central nuclei (Figures 4, 5C). No main effects of Age or TOD were observed in the amygdala. Together, these findings suggest that constitutive *Arc* expression levels vary across age in the hippocampus and undergo diurnal regulation in the hippocampus and visual cortex differentially across age groups.

#### Developmental stage and TOD interact to control Arc expression in the amygdala but not the hippocampus

For maze-exposed animals, *Arc* expression measures were normalized to the mean baseline values from the appropriate age and TOD-matched controls. No effects of CX614 or illumination level were observed in overarching four-way ANOVAs (age × drug × lighting × TOD). As such, data were collapsed across drug doses and illumination levels and two-way ANOVAs (Age × TOD) were performed. For the amygdala, we observed a main effect of TOD (*F*_(1,12)_ = 14.047, *p* = 0.003), in addition to an Age × TOD interaction (*F*_(1,12)_ = 5.126, *p* = 0.043) (Figure 5A). *Post hoc* examination showed a within-age effect as the number of *Arc*-positive neurons at P17–19 was increased in the PM compared to the AM (Tukey HSD: *p* = 0.003) and a near significant across-age effect reflecting more *Arc*-positive neurons at P22–24 compared to P17–19 when testing was performed in the AM (Tukey HSD: *p* = 0.053). Neither age nor TOD influenced* Arc* expression in the hippocampus of EPM-exposed rats (Figure 5B). These findings support the idea that, with increasing age at the end of the third postnatal week, anxiety-induced neuronal activation becomes less sensitive to TOD selectively in the amygdala.

#### Developmental stage and environmental variables interact to control Arc expression in layer 4 but not layer 2/3 of the primary visual cortex

There was no Age nor TOD effect on *Arc* expression in layer 4 of V1 (Figure 5D). In layer 2/3, there was a main effect of Age (*F*_(1,13)_ = 6.083, *p* = 0.036) reflecting greater *Arc* expression levels at P17–19 (*n* = 7) compared to P22–24 (*n* = 10). There was also an Age × TOD interaction (*F*_(1,13)_ = 5.922, *p* = 0.038) and a nearly significant effect of TOD (*F*_(1,13)_ = 4.608, *p* = 0.06) on number of *Arc*-positive neurons in layer 2/3 (Figure 5E). In the AM, *Arc* expression was greater at P22–24 compared to P17–19 (Tukey HSD: *p* = 0.049). *Arc* expression was also greater at P22–24 in the AM compared to the PM (Tukey HSD: *p* = 0.044). These results show subregional sensitivities of the primary visual cortex to EPM exposure that are age- and TOD-dependent.

## Discussion

Assessment of EPM behavior in late postnatal development revealed age-related differences in innate anxiety with greater temporal resolution than shown in previous experiments. In accord with prior studies examining the effects of age on EPM performance (Imhof et al., 1993; Andrade et al., 2003), open arm dwell time was reduced with increasing age from P17–19 to P22–24, supporting the notion that animals under 3 weeks of age experienced less anxiety on the EPM. Interestingly, at P17–19, open arm exploration occurred primarily under bright illumination in the AM, while dim illumination encouraged the greatest open arm times at P22–24. Similar to prior reports (Blair et al., 2013), CX614 delivery did not affect anxiety behavior at either testing age. Combined, the results suggest that illumination levels and testing TOD can produce opposing effects on anxiety behavior depending on postnatal age.

In parallel, mechanisms governing stress responsiveness varied as a function of developmental stage. In behaviorally naïve animals, elevated CORT levels were measured at P22–24 compared to P17–19, regardless of TOD. However, CORT levels increased more at P17–19 compared to P22–24 in maze-tested rats, suggesting greater stress reactivity at P17–19 or a ceiling effect at P22–24 due to higher baseline CORT levels. Diurnal differences in CORT concentrations were coupled with open arm exploration on the EPM at both ages, such that both CORT levels and open arm exploration were highest in the AM and declined across the day. However, only at P22–24 was CORT concentration directly and negatively correlated TOD (binned by hour). Corroborating prior reports that delineate the developmental time course for CORT rhythmicity to be around 3 weeks of age (Takahashi et al., 1979; Honma and Honma, 1986), the current data reveal more mature diurnal regulation of CORT responsiveness to EPM exposure at P22–24 but not P17–19.

*Arc* is an activity-dependent immediate early gene that is rapidly expressed in neurons in response to processes such as spatial learning and memory and fear conditioning (Guzowski et al., 2001; Czerniawski et al., 2011), and serves as an important marker for neuronal activation and plasticity (Guzowski et al., 1999; Okuno, 2011). It is important to note that, while moderate behavioral stress increases *Arc* expression in the basolateral amygdala of adult male rats, more severe behavioral stress, well beyond the level experienced during EPM exposure, was found to be required to negatively impact *Arc* expression (Ons et al., 2004) Therefore, it is reasonable to infer neuronal activity levels from the degree of *Arc* expression in response to EPM exposure. Comparisons were not made across structures due to variation in the time course for *Arc* expression (Guzowski et al., 1999; Huff et al., 2006; Shepherd and Bear, 2011). Overall, *Arc* expression levels in area CA1 of the hippocampus and layer 4 of V1 were not different between age groups. However, age- and TOD-related differences emerged in the amygdala and layer 2/3 of V1. In the amygdala, more *Arc*-positive neurons were observed in the PM vs. AM at P17–19 and more *Arc*-positive neurons were observed at P22–24 compared to P17–19 during AM testing. In layer 2/3 of area V1, more *Arc*-positive neurons were observed at P22–24 in the AM and more *Arc*-positive neurons were observed at P17–19 in the PM. Thus, age effects on levels of *Arc* expression in the amygdala tended to be inversely related to developmental differences in open arm time while *Arc* expression in the visual cortex more directly agreed with the effects of age on open arm time. Overall, differences in neuronal activation patterns across brain structures in response to EPM exposure at different testing ages further implicate varied perceptual and emotional influences over EPM performance. Future studies involving blockade of CORT receptors might clarify if the correspondence between plasma CORT levels and amygdala or cortical *Arc* expression levels was causal or correlational.

Many studies have revealed an increase in plasma CORT following exposure to the EPM (Andreatini and Leite, 1994; File et al., 1994; Sütt et al., 2010). Some reports indicate an inverse relationship between plasma CORT and open arm time (Albrechet-Souza et al., 2007; Mitra and Sapolsky, 2008), while others show no relationship between plasma CORT level and behavioral measures on the EPM in adult animals (Rodgers et al., 1999; Butler et al., 2014). We found no direct correlation between CORT level and open arm time in juvenile rats. Rodgers et al. (1999) have shown that CORT levels increase in response to near attempts to enter the open arms rather than actual exploration of them, and elevated CORT is correlated with risk assessment but not locomotion in adult animals. The lack of an explicit relationship between open arm time on the EPM and either CORT or amygdala *Arc* expression in our data corroborates reports by Rodgers and colleagues that biological responses to EPM exposure are not necessarily directly correlated with time spent on open arms of the maze, and extends this rationale to juvenile animals.

Differential TOD effects on anxiety-like behavior at P17–19 and P22–24 may be explained by the late postnatal development of circadian rhythmicity. While rhythmic activity-rest cycles across the day are present as early as P10 (Reppert et al., 1984), it is not until after 3 weeks of age that circadian activity rhythms are entrained to the light cycle (Honma and Honma, 1986) so that by 2 months of age, peak activity occurs during the dark phase (Andrade et al., 2003). In developing rats, before the circadian shift occurs, locomotor activity is highest during the first quartile of the day, compared to heightened locomotion in the third quartile in older animals (Smith and Morrell, 2011). Our findings agree in that young animals explore the EPM most during the AM, and only P22–24 rats exhibit diurnal differences in stress reactivity. These findings suggest that, to some extent, proclivity for locomotion may offset anxiety level to increase open arm exploration.

One reason for differential influences of illumination across ages might be attributed to disparities in visual acuity. Studies have shown that when transparent railings on the open arms are covered by colored paper, exploration of those arms is significantly higher, suggesting that visual perception impacts risk assessment in open spaces (Martìnez et al., 2002). Many adult-like properties of visual capacity are present by P23, though maturity of all tested visual abilities was not apparent until P45 (Fagiolini et al., 1994). Indeed, at P18, rats spend ten times longer than adults exploring the brightly lit portion of a two-chambered box (Smith and Morrell, 2007). However, this difference disappears with maturation such that, by 30 days of age, performance was similar to adults (Slawecki, 2005). Thus, at P17–19, rats may navigate open areas of the EPM more under bright illumination because these conditions permit enhanced visual perception. As sensory abilities improve, performance more closely resembles that of adults and dim illumination encourages the greatest exploration of open maze areas.

Since the way in which animals explore their environment varies as a function of maturity level (File, 1978; Weinert, 2000), it is not surprising that the first month of life has been characterized by the greatest variability in anxiety-like behavior in rodents (Andrade et al., 2003). Age-related differences in biological and behavioral variables during early development are manifested through variations in exploratory mechanisms; particularly, an increased drive to explore novel places as well as an under-developed risk assessment capacity in young rats (Laviola et al., 2003; Doremus et al., 2006). Exposure to novelty prior to EPM testing has been shown to be anxiolytic (Darwish et al., 2001), and it has been suggested that bright lighting may have a similar potential to act as a novel stimulus in adolescents (Slawecki, 2005). In this regard, the profound increase in anxiolytic behavior observed at P17–19 under bright illumination may reflect increased novelty-seeking behavior elicited by the bright light that blunts effects of anxiety.

Claims have been made that inclusion of the center square in the scoring of EPM open arm time is inappropriate because it does not directly gauge anxiety (for reviews, see Hogg, 1996; Rodgers and Dalvi, 1997; Carobrez and Bertoglio, 2005). However, time spent exploring the center square has been suggested to reflect decision-making underlying approach/avoidance conflict and risk assessment (Rodgers and Dalvi, 1997). The current results demonstrate that inclusion of time in the center square is particularly useful when assessing anxiety behavior in developing rodents. In fact, the observed age differences in open arm time were carried entirely by the time in the center square. These findings align with conclusions made by Smith and Morrell (2011) that late preweanling rats display differential sensitivities to environmental stimuli than adults, while at the same time expressing reduced fear of open spaces, even within the home cage (Smith and Morrell, 2007). In general, the findings support the idea that behavior of juvenile rats on the EPM results from overlapping developmental trajectories for multiple neural systems involved in sensorimotor ability, anxiety, and risk-assessment.

In summary, the current findings accentuate differences in the processing of and response to anxiety-provoking stimuli according to postnatal age, suggesting that biological and behavioral mechanisms supporting anxiety genesis are underdeveloped prior to 3 weeks postnatal. We show that environmental parameters such as illumination level and TOD differentially influence anxiety responses on the EPM, likely through engagement of diverse regulatory mechanisms across developmental stages. Overall, behavior of juvenile rats under 3 weeks of age seems to be driven more by novelty exploration, possibly due to underdeveloped visual and risk assessment capacities. Interestingly, less than a week later, behavior more closely resembles adults and is associated with parallel changes in endocrine and neural activation patterns across testing conditions. These results show that methodological considerations are particularly important in studies of behavioral maturation since various components of an animal’s environment can impact maze behavior in non-uniform ways. The findings illustrate that continued growth and plasticity of the juvenile brain in the late postnatal period imposes difficulties in providing neural explanations for behavioral modifications at this developmental stage, and may have implications for better understanding childhood anxiety disorders.

## Authors contribution

Theodore Dumas and Sarah Albani designed the study. Sarah Albani, Marina Andrawis and Rio Jeane Abella, collected behavioral and CORT data. Sarah Albani, Marina Andrawis, Rio Jeane Abella, John Fulghum and Nagmeh Vafamand collected ISH data. Sarah Albani, Marina Andrawis, and Rio Jeane Abella analyzed the data. Theodore Dumas and Sarah Albani wrote and edited the manuscript.

## Conflict of interest statement

The authors declare that the research was conducted in the absence of any commercial or financial relationships that could be construed as a potential conflict of interest.
